# Supplementary material for: iTRAQ-Based Proteomic and Physiological Analyses Reveal the Mechanisms of Dehydration and Cryopreservation Tolerance of Sophora tonkinensis Gagnep. Seeds
Source: Plants (Basel). 2023 Apr 29;12(9):1842. doi: 10.3390/plants12091842 (PMC10180571; doi:10.3390/plants12091842)
Supplement: Supplementary file 1 [file plants-12-01842-s001.zip › Supplementary Figures.pdf]

plants

Supplementary Materials

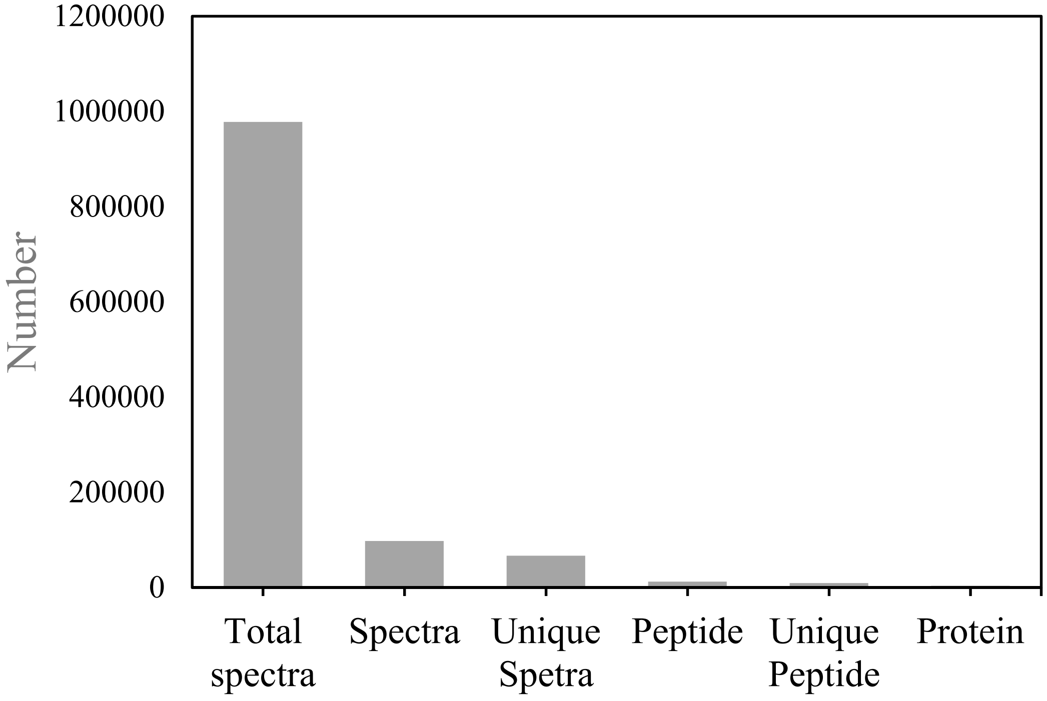

Figure S1. Spectra, peptides, unique peptides, and proteins identified from iTRAQ proteomics after searching against the Sophora tonkinensis databases.

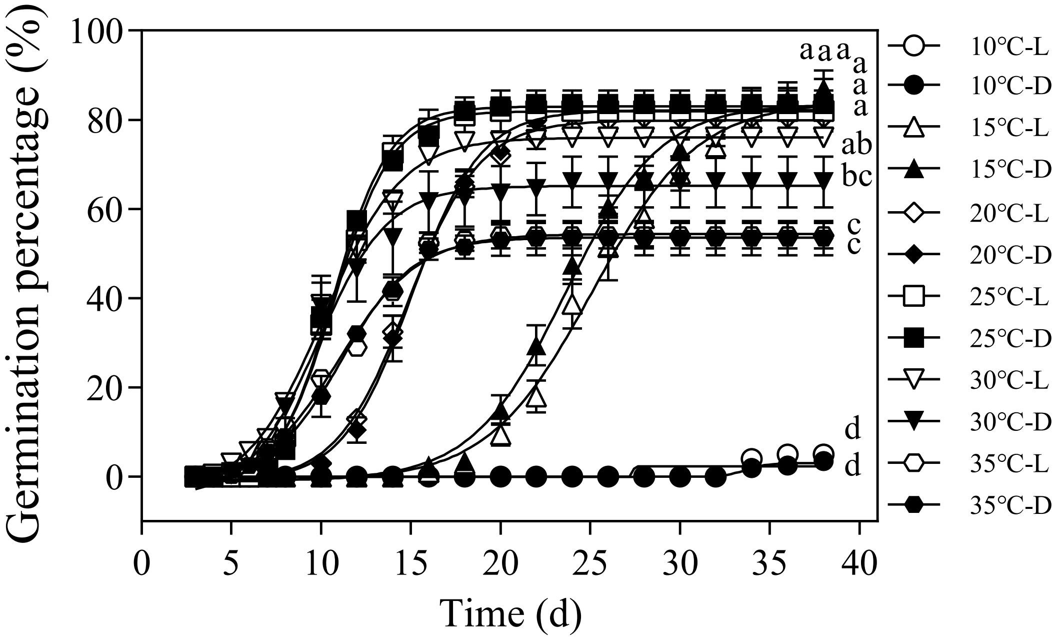

Figure S2. Germination process of fresh mature Sophora tonkinensis seeds under constant temperature and alternating light / dark conditions (L is the light condition and D is the dark condition).
